# Perceived Target Range Shapes Human Sound-Localization Behavior

**DOI:** 10.1523/ENEURO.0111-18.2019

**Published:** 2019-04-05

**Authors:** Rachel Ege, A. John Van Opstal, Marc M. Van Wanrooij

**Affiliations:** Department of Biophysics, Radboud University, Donders Institute for Brain, Cognition and Behaviour, 6525 AJ Nijmegen, The Netherlands

**Keywords:** auditory system, Bayes, endogenous, head movement, learning, models

## Abstract

The auditory system relies on binaural differences and spectral pinna cues to localize sounds in azimuth and elevation. However, the acoustic input can be unreliable, due to uncertainty about the environment, and neural noise. A possible strategy to reduce sound-location uncertainty is to integrate the sensory observations with sensorimotor information from previous experience, to infer where sounds are more likely to occur. We investigated whether and how human sound localization performance is affected by the spatial distribution of target sounds, and changes thereof. We tested three different open-loop paradigms, in which we varied the spatial range of sounds in different ways. For the narrowest ranges, target-response gains were highly idiosyncratic and deviated from an optimal gain predicted by error-minimization; in the horizontal plane the deviation typically consisted of a response overshoot. Moreover, participants adjusted their behavior by rapidly adapting their gain to the target range, both in elevation and in azimuth, yielding behavior closer to optimal for larger target ranges. Notably, gain changes occurred without any exogenous feedback about performance. We discuss how the findings can be explained by a sub-optimal model in which the motor-control system reduces its response error across trials to within an acceptable range, rather than strictly minimizing the error.

## Significance Statement

Sensory observations can be noisy, leading to uncertainty in perceptual inferences and variable estimation errors. Theoretically, to reduce uncertainty, sensory information could be integrated with knowledge from prior experience, and with feedback about one’s own response behavior. Here we show, that for a basic and accurate sensorimotor task such as sound localization, humans indeed rely on perceived experience in the absence of exogenous feedback, as they rapidly changed their response sensitivity to experimental variations in the spatial distribution of targets. We argue that the auditory system reduces its estimated localization error close to its expected minimum across trials, allowing for idiosyncratic sub-optimal target response gains.

## Introduction

To localize sounds, the auditory system relies on interaural time and level differences, which vary systematically in the horizontal plane (azimuth; Blauert, 1997), while the pinnae provide spectral-shape cues by diffracting and reflecting sound waves for directions in the median plane (elevation; Middlebrooks and Green, 1991; Kulkarni and Colburn, 1998; Hofman et al., 1998; Bremen et al., 2010). Under simple free-field laboratory conditions, the acoustic cues enable humans to accurately localize sounds in all directions (Middlebrooks and Green, 1991; Wightman and Kistler, 1989).

However, natural environments typically contain an unknown number of sound sources, and the neural processing may be endowed with internal noise and uncertainty, rendering the auditory system prone to localization errors (Hofman and Van Opstal, 1998; Langendijk and Bronkhorst, 2002). To minimize such errors, the nervous system should not only rely on immediate sensory evidence, but also acquire information about the environment. Such strategies have been demonstrated for perceived visual motion (Stocker and Simoncelli, 2006), visuomotor integration (Körding and Wolpert, 2004), movement planning (Hudson et al., 2007), audiovisual integration (Alais and Burr, 2004), and multisensory cue combination (Körding et al., 2007).

What follows is a brief explanation of what error minimization actually entails when generating a response R toward a perceived sound presented at target location T. The response R will be guided by the target T, but is also affected by internal additive noise (ε), due to a noisy sensory observation of the target and/or a noisy motor response. This can be well-described with a linear equation (Goossens and Van Opstal, 1999; Van Wanrooij and Van Opstal, 2005; Van Grootel et al., 2011; Van Barneveld and Van Wanrooij, 2013; Ege et al., 2018):

(1) $$R=g\cdot \left(T+\varepsilon \right)$$

with g the response gain (slope). In the absence of noise, the optimal behavior is described by R = T, with a gain of 1. Over *N* trials, the mean absolute localization error is determined by:

(2) $$\overset{¯}{E}=\frac{\sum_{n=1}^{N}\left|R_{n}-T_{n}\right|}{N}=\frac{\sum_{n=1}^{N}\left|\left(g-1\right)\cdot T_{n}+g\cdot \varepsilon_{n}\right|}{N}$$

**Figure 1.**
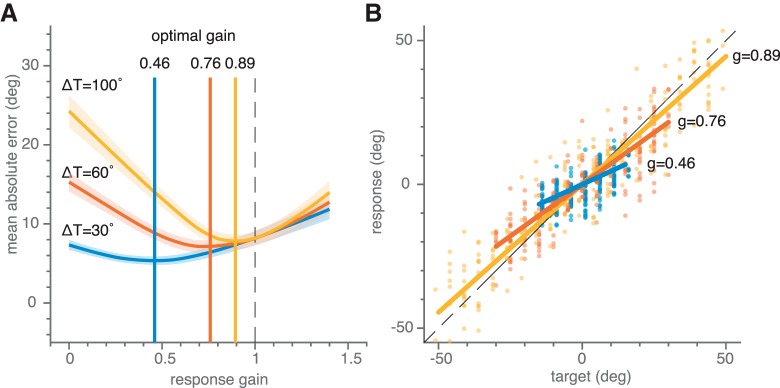
Model simulations showing that error minimization leads to an optimal target-response gain <1. ***A***, Mean absolute error (Eq. 2) as a function of the response gain for three different target ranges [ΔT±50° (yellow), ΔT±30° (red), and ΔT±15° (blue)], with additive, p(ε) = N(0,σ_ε_) Gaussian noise. Simulations were obtained by uniformly randomly picking 200 target locations from each target range and generating responses according to Equation 1 for 141 gains g ranging from 0 to 1.4 with a fixed additive noise standard deviation of 10.0°. The mean absolute error is determined for every simulation according to Equation 2. The simulation was repeated 1000 times for each gain, to obtain the average (indicated by bold colored curves) mean absolute error and its standard deviation (indicated by the colored patches). The minimum average mean absolute error is obtained for gains <1. The optimal gains systematically vary with target range (vertical lines). The highest optimal gain (*g* = 0.89) is found for the largest target range, for which the absolute error varies strongest with gain. ***B***, Single simulations of stimulus-response relations (Eq. 1) for three target ranges at their respective optimal response gains.

From this follows that the mean absolute error depends on localization accuracy which is highest if the gain is one [as captured by the systematic error term $\left(g-1\right)\cdot T_{n}$ being reduced to 0°]; and on localization precision, which is highest if the gain is zero (minimizing the random error term $g\cdot \varepsilon_{n}$). To minimize its errors, the audiomotor system should therefore optimize accuracy-precision trade off (see also Ege et al., 2018). This would typically be obtained for a gain *g* < 1; with the exact value also depending on the extent of the spatial target range (Fig. 1). Essentially, gain optimization requires knowledge about the amount of one’s own response variability and about the likely source locations of targets.

But how does the auditory system access such information without independent feedback (e.g., visual)? We hypothesize that the system could employ two sources of information under open-loop localization tasks: the acoustic cues to estimate perceived sound-source locations, and internal neural feedback about orienting movements that provide information about its responses. Sensorimotor integration could thus provide a neural estimate of the system’s overall performance, which could lead to potential adjustments in the response gain, even in the absence of exogenous feedback. Thus, if the perceived distribution of sounds differs from the system’s priors, it could adjust the response gain to minimize its internal estimate of sound localization errors.

In three experiments, we investigated how listeners incorporate the perceived target-distribution range in their localization responses. The first experiment tested whether the target range influenced the response gain, by presenting fixed spatial ranges that varied between subsequent blocks of trials. We found that this is indeed the case, irrespective of the order of the blocks. The second experiment tested the adaptive capacity of the response gain, by presenting a long block of trials with a step-change (either upward, or downward) in the target range halfway the block. We observed a rapid gain change that differed for upward versus downward step changes, as well as slow gain changes before and after the step. In the third experiment, we studied how the gain responds to a continuous change in the target range at different speeds. We discuss our results within the context of models for sensorimotor integration.

## Materials and Methods

### Participants

We collected data from twelve participants (seven male) who took part in three experimental paradigms (experiment 1: eight participants; experiment 2: 10 participants; experiment 3: seven participants; see below, Paradigms). Six subjects (S1–S6) participated in all three paradigms. All participants had normal or corrected-to-normal vision, and no reported hearing dysfunctions, aged 21–31 (mean, 26.6 years). One participant (S1) is author of this paper; the other eleven participants were naive about the purpose of this study. Experiments were conducted after obtaining informed consent from the participant.

The experiments fully adhered to the protocols regarding observational experiments on healthy human adults and were approved by the local institutional ethical committee of the Faculty of Social Sciences at the Radboud University (ECSW 2016-2208-41). All participants signed an informed consent form, before the start of the experimental sessions.

### Apparatus

During the experiment, the subject sat comfortably in a chair in a completely dark, sound attenuated room (L × W × H = 3.5 × 3.0 × 3.0 m). The floor, ceiling and walls were covered with sound-attenuating black foam (50 mm thick with 30-mm pyramids; AX2250, Uxem BV), effectively eliminating echoes for frequencies exceeding 500 Hz. The room had an ambient background noise level of ∼30 dBA (measured with an SLM 1352P, ISO-TECH sound-level meter). The chair was positioned at the center of a spherical frame (radius 1.5 m) on which 125 small broad-range loudspeakers (SC5.9; Visaton GmbH) were mounted. These speakers were organized in a grid by separating them from the nearest speakers by an angle of ∼15° in both azimuth and elevation according to the double-pole coordinate system (Knudsen and Konishi, 1979). On the cardinal axes (elevation zero, and azimuth zero) speakers were placed more densely; these were separated by 5°. No speakers were placed at elevations below –45°. Head movements were recorded with the magnetic search-coil technique (Robinson, 1963). To this end, the participant wore a lightweight spectacle frame with a small coil attached to its nose bridge. Three orthogonal pairs of square coils (6-mm^2^ wires, 3 × 3 m) were attached to the room’s edges to generate the horizontal (80 kHz), vertical (60 kHz), and frontal (48 kHz) magnetic fields, respectively. Horizontal and vertical head-coil signals were amplified and demodulated (EM7; Remmel Labs), low-pass-filtered at 150 Hz (custom built, fourth-order Butterworth), digitized by a Tucker Davis Technologies (TDT, RRID:SCR_006495) System 3 Medusa head stage and base station (RA16GA and RA16, respectively), and stored on hard disk at 6 kHz/channel. Custom-written MATLAB (RRID: SCR_001622) software, running on a PC (HP EliteDesk) controlled data recording, stimulus generation, and online data visualization.

### Stimuli

Acoustic stimuli were digitally generated using TDT hardware, consisting of two real-time I/O data acquisition processors (RP2.1, at a 48,828.125-Hz sampling rate), two stereo amplifiers (SA-1), four programmable attenuators (PA-5), and eight multiplexers (PM-2). Each of the 100 available acoustic stimuli consisted of 50 dB (A-weighted), 50-ms duration, pre-generated fresh Gaussian white noise (0.5- to 20-kHz bandwidth), with 5-ms sine-squared onset and cosine-squared offset ramps.

Visual stimuli consisted of green LEDs (wavelength 565 nm) mounted at the center of each speaker (luminance 1.4 cd/m^2^), which served as independent visual fixation stimuli during the calibration experiment, or as a central fixation stimulus at straight-ahead during the localization experiments.

### Calibration experiment

To establish the off-line mapping of the coil signals onto known target locations subjects pointed a laser, attached to the spectacle frame, toward 24 known LED locations in the frontal hemifield (separated by ∼30° in both azimuth and elevation).

### Paradigms

In all paradigms, participants were instructed to first fixate the central LED by aligning the head-fixed laser pointer. The fixation light was extinguished 300–800 ms after a button press of the participant and 200 ms later the target sound was presented. Participants were instructed to “point the head-fixed laser as fast and as accurately as possible toward the perceived location of the sound source”. Data acquisition ended automatically 1500 ms after sound onset, after which a new trial was initiated. Inter trial intervals arising from processing time to end a trial (e.g., data storage on disk) and initiate a new trial (e.g., loading new sound in TDT) lasted on average 2 s. Onset of one trial to onset of the next trial took on average 4 s.

**Figure 2.**
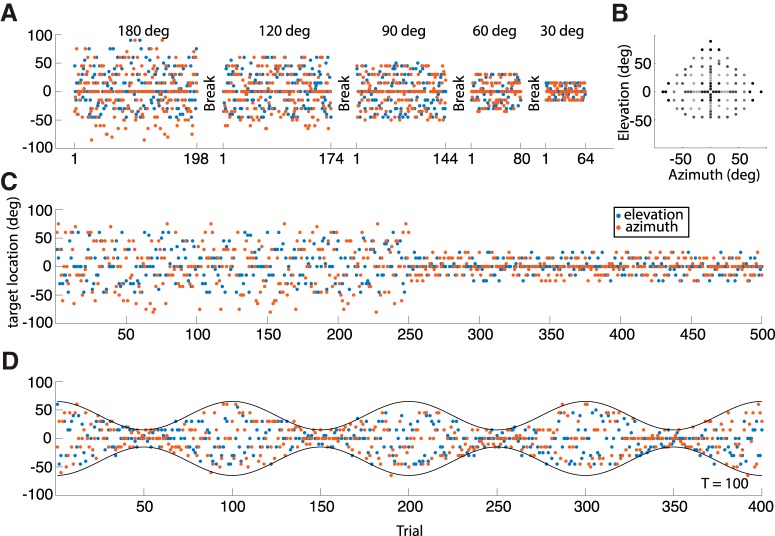
Experimental paradigms. ***A***, ***C***, ***D***, Colored dots indicate stimulus positions, for azimuth (red) and elevation (blue), as a function of trial number. ***A***, Experiment 1: five target blocks, shown in descending order of target-range. ***B***, Distribution of all speakers in the experimental room in double-pole azimuth-elevation coordinates. ***C***, Experiment 2: after 250 trials, the stimulus range acutely changed from a large (±55°) to a small (±25°) range (as shown), or vice versa. ***D***, Experiment 3: the stimulus range changed in a sinusoidal way throughout the experiment (400 trials) from large (±60°) to small (±15°), or vice versa. The panel shows a repetition period *p* = 100 trials, and phase ϕ = 0.

Subjects participated in three experimental paradigms with varying ranges for the target sound locations, as detailed below (Fig. 2). Sound locations were pseudo-randomly selected from a discrete uniform distribution over all speakers within the experimental range (Fig. 2*B*). The actual realization of locations and presentation order was fixed before the start of the study and was the same for all participants. Participants received no information about the stimulus distribution ranges, and they were not told about the potential changes in the target distribution. Experiments were performed under open-loop hearing conditions, as participants did not receive any feedback about their performance during, or after the experiment. Note that the stimuli within the smallest range in each of the experiments were the same for all experimental blocks, although their relative occurrence decreased with increasing target range.

#### Experiment 1

In the first experiment (Fig. 2*A*), the range of stimulus locations was kept constant within a block of trials but varied across blocks. We presented five different ranges as blocks of trials to eight participants (four male; aged 27–31, mean: 28.3 years; S1–S8):

(1) ΔT = 30° (±15° in azimuth and elevation), 16 locations, each presented four times, yielding a total of *N* = 64 stimuli (Fig. 2*A*, far right),
(2) ΔT = 60°, 40 locations, *N* = 80 stimuli (Fig. 2*A*, 2nd panel from right),
(3) ΔT = 90°, 72 locations, *N* = 144 stimuli, in two parts (Fig. 2*A*, 3rd panel from right),
(4) ΔT = 120°, 87 locations, *N* = 174 stimuli, in two parts (Fig. 2*A*, 2nd panel from left),
(5) ΔT = 180°, 99 locations, *N* = 198 stimuli, in two parts (Fig. 2*A*, far left).

**Figure 3.**
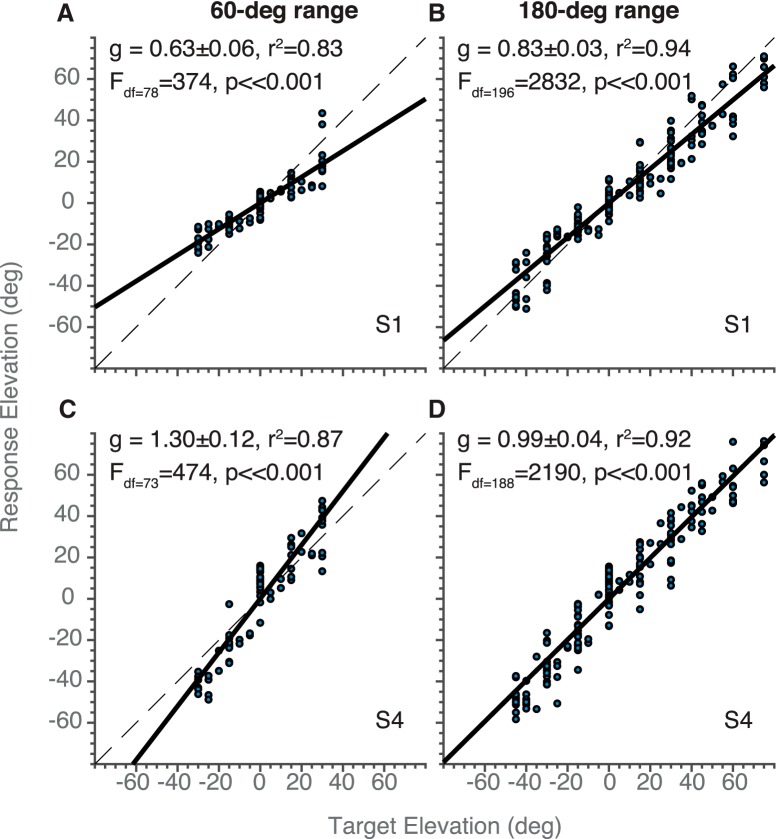
Example stimulus-response plots for experiment 1. Stimulus response plots in elevation for participant S1 (***A***, ***B***) and S4 (***C***, ***D***), for the (***A***, ***C***) 60° and (***B***, ***D***) 180° target-range blocks presented in decreasing order. Filled circles denote individual localization responses, the black solid line represents the best-fit regression line (Eq. 4), with *g* the response gain of the fit; the dashed lines indicate the perfect stimulus-response relation (x = y). The insert text depicts the fitted gain, *g*, including its 95% confidence interval, the *r^2^* between data and fit, and the *F* and *p* values for the linear fit, including the degrees of freedom.

The five blocks were presented within one experimental session, with short intermittent breaks (∼2 min), during which the lights were turned on. In three sessions, stimulus blocks within a session were sorted either by increasing order in target range, from ΔT = 30–180°, by decreasing order in target range, from ΔT = 180–30° (as in Fig. 2*A*), or pseudo-randomly. Completion of a session of 660 trials took ∼50 min.

#### Experiment 2

In the second paradigm, the distribution range of target locations switched after the first half of the experiment from ΔT = 110° (±55°, *N* = 250 trials) to ΔT = 50° (±25°, *N* = 250 trials; broad-to-narrow; Fig. 2*C*) in one session, and vice versa (narrow-to-broad) in a second session. Ten listeners (five male, aged 21–29, mean: 26 years; S1–S6, S9–S12) participated in both sessions, with a different order of range switching. These sessions were held on two separate days. There were no interleaved breaks within a session. One session of 500 trials took ∼35 min.

#### Experiment 3

In the third paradigm (Fig. 2*D*), the range of stimulus locations varied dynamically following a sinusoidal envelope with one of four periods, P (in number of trials), centered around straight ahead, according to:

(3) $$\Delta T_{n}=75\cdot \left(1+0.6\cdot \cos\left(2\pi \frac{n}{P}+\phi \right)\right)$$

with trial number *n* = [0:399], and period *p* = [50, 100, 200, 400] trials. A session could either start at the maximum range of ΔT_max_ = 120° (Fig. 2*D* shows *p* = 100, ϕ = 0) or at the minimum range of ΔT_min_ = 30° (ϕ = π). The seven subjects (three male; aged 27–29, mean: 28 years; S1–S6, S8), who participated in this experiment, completed all eight conditions (four frequencies × two phases, divided over eight sessions of 400 trials each). There were no interleaved breaks. One session took ∼26 min.

### Analysis

The head-position signals (in Volts) were first digitally low-pass filtered (cutoff frequency 75 Hz, filter order 50) and calibrated (to degrees of head rotation from center). A custom-written MATLAB program detected head-movement onsets, whenever the velocity first exceeded 20°/s, and offsets when they first fell below 20°/s after a detected onset. We took the end position of the first movement after stimulus onset as a measure for localization performance and excluded potential secondary corrective movements. Each movement-detection marking was visually checked by the experimenter, and adjusted when deemed necessary, without having information about the stimulus. Data analysis and visualization were performed in MATLAB.

### Statistics

The optimal linear regression line of the stimulus-response relation was determined by minimizing the sum-squared deviation of:

(4) $$R_{n}=b+g_{exp}\cdot T_{n}$$

The dimensionless slope, *g_exp_* (with experiment = 1, 2, or 3), or gain, of Equation 4 quantifies the sensitivity (resolution) of the responses to changes in target position; the offset, *b* (in degrees), is a measure for the listener’s response bias. A perfect localization response would have a gain of 1.0° and a bias of 0.0°, irrespective of the experimental conditions. Given the rationale of this study (see Introduction), we took the response gain as the relevant parameter that could potentially change with the imposed changes in the experimental target range. The response bias b was always negligible (close to 0°), and is not further studied here.

#### Experiment 1

For the first paradigm (Fig. 2*A*), the experimental variable of main interest was the target range, ΔT, which was kept fixed within a block, but differed between blocks. In first approximation, we describe how the gain depends on the target range through a linear relation, with two free parameters:

(5) $$g_{I}=\beta_{0}+\beta_{1}\cdot \frac{\Delta T}{180}$$

(normalized with respect to the maximum target range of ΔT = 180°). Thus, Equation 4 becomes:

(6) $$R_{n}=b+\left(\beta_{0}+\beta_{1}\cdot \frac{\Delta T_{k}}{180}\right)\cdot T_{n}$$

Here, we denoted parameter $\beta_{0}$ as the gain intercept, which can be interpreted as the subject’s default (prior) gain in the absence of any target information, and $\beta_{1}$ as the gain slope, which measures how the response gain changes as a function of the target range.

#### Experiment 2

In the second experiment, the experimental variable of main interest was trial number. We again took a first-order approximation to describe how the gain might depend on trial number. To that end, the data from the two long half-blocks in the experiment were fitted separately: before (trials *n* = 1–250) and after (trials *n* = 251–500) the step-change in the target range, with a gain according to:

(7) $$g_{II}=\beta_{0}+\beta_{1}\cdot \left(\frac{n}{250}-k\right)$$

Now, $\beta_{0}$ is called the “initial gain,” measured at the start of each sub-block (either at the beginning of the session, or immediately after the switch), and $\beta_{1}$ is the gain-slope, as above (with k = 0 for the first half-block, or k = 1 for the second half-block). Thus, for the analysis of this experiment, we reformulated Equation 4 as:

(8) $$R_{n}=b+\left(\beta_{0}+\beta_{1}\cdot \left(\frac{n}{250}-k\right)\right)\cdot T_{n}$$

According to these definitions, the “narrow-range gain at the switch” and the “gain change at the switch” are determined by $\beta_{0},\beta_{1}$, and by the switch direction (small to large vs large to small target range; see Fig. 6*B*).

#### Experiment 3

For the third experiment, the experimental variable of main interest was the trial period. Here, we assumed that the instantaneous gain would vary in a sinusoidal way with the instantaneous trial number, normalized for the period:

(9) $$g_{III}=\beta_{0}+\beta_{1}\cdot \sin\left(2\pi \frac{n}{P}+2\pi \varphi \right)$$

Thus, in this case, the regression analysis of Equation 4 becomes:

(10) $$R_{n}=b+\left(\beta_{0}+\beta_{1}\cdot \sin\left(2\pi \frac{n}{P}+2\pi \varphi \right)\right)\cdot T_{n}$$

with *n* trial number (where *n* = 0 is defined as the first trial from the largest target distribution, and *n* = P/2 as the first trial from the narrowest distribution). In Equation 10, $\beta_{0}{-\beta }_{1}$ corresponds to the response gain for the narrow target range, while 2β_1_ is the total gain change in the experiment.

All fits to the models of Equations 6, 8, 10 were obtained by least-square-error procedures with robust bisquared weighing options in MATLAB. We determined Pearson’s linear correlation coefficient, *r*, between model prediction and response, and *r^2^*, which is the coefficient of determination (a measure for the goodness of fit of the applied model, or the explained variance of the data). As these values were typically high (mean *r^2^* was 0.92, and each *r^2^* was highly significant, all *p* ≪ 0.001), we asserted that these models provided an adequate description of the data.

For each parameter obtained, we also determined the 95% confidence interval.

The results suggested that both gain parameters ($\beta_{0},\beta_{1}$) in Equations 6, 8, 10 were correlated. To test that, simple linear regression was performed, and the slope, goodness-of-fit *r^2^*, *F* statistic and corresponding *p* value were obtained.

### Windowing

**Figure 4.**
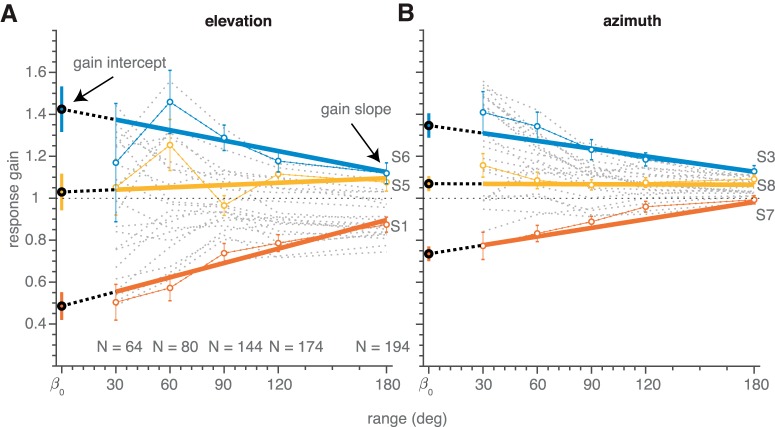
Gain dependence on target range in Experiment 1. Localization gains for all subjects (gray dotted lines) for elevation (***A***) and azimuth (***B***) components determined for each target range. Connected colored open circles denote the localization gains for three representative subjects; error bars indicate the 95% confidence interval. Bold colored lines denote the best fit regression lines of Equation 6 through the data of these subjects. Color-filled circles on the ordinate indicate the gain intercepts (β_0_; Eq. 6).

For illustrative purposes, we also performed regressions on non-overlapping windowed sections of the data (Figs. 4, 6, 8, light-gray lines). In experiment 1, the response gain was supposed to vary with target range. The windows thus constituted the different blocks, which were analyzed separately with the linear regression analysis of Equation 6 (data from the 120° and 180° target ranges were pooled).

In experiment 2, the response gain was supposed to depend on trial number. The 500 trials were divided into ten windows of 50 trials, on which separate regression analyses were performed.

In experiment 3, the response gain was supposed to depend on instantaneous trial number. After normalizing for the period (and aligning the data from blocks starting with a large, or a small range), the oscillation period *P* was divided into 11 windows of equal size, on which separate regressions were performed. Note that the first and last window of a period contained the same data.

## Results

### Localization gain changes

In the first experiment, subjects oriented to sounds drawn from five different spatial target distributions, presented in separate blocks (Fig. 2*A*). The rationale of this design was (Fig. 1), that if humans were to integrate information about the perceived spatial target range with their sensory-motor observation of a current target, the measured response gains toward the same stimulus might vary for the different target ranges.

Figure 3 shows four examples of the stimulus-response behavior of the elevation components of goal-directed head-movements for two participants (S1 and S4), each confronted with two different target ranges, ΔT = 60 (Fig. 3*A*,*C*) and ΔT = 180° (Fig. 3*B*,*D*), respectively, and presented in the decreasing range order. Note that the response variability (i.e., variance of the residuals; the inverse of precision) across conditions and subjects was quite comparable, as evidenced by *r^2^* values around 0.9. However, both subjects display different response patterns regarding their accuracy: whereas the head movements of S1 had considerable target undershoots for the 60° target range, as measured by the relatively low response gain (*g* = 0.63), subject S4 tended to generate overshoots for these same targets (*g* = 1.30). For the 180° target range, however, both subjects had adjusted their response gains to values that were closer to the ideal value of *g* = 1.0. Indeed, the change in response gains for the 180° target range with respect to the 60° target range was considerable: *Δg* = +32% for S1, and *Δg* = –24% for S4.

The linear-regression results of Figure 3 are exemplary for the response behavior across all eight subjects, irrespective of the order in which the stimulus ranges were presented (see Materials and Methods). To illustrate this important aspect of the data, we plotted the response gains obtained from the regression analyses for the five different target ranges, the three different range orders, for all subjects in Figure 4 as a function of the target range. It is immediately clear that the intersubject variability in response gains across subjects for the small target ranges was much larger than for the largest target range, for both response components. In other words, subjects with large overshoots to targets in the small range systematically decreased their response gain with increasing target range (like S4 in Fig. 3). In contrast, subjects with large undershoots to targets in the small range increased their gain with target range (like S1 in Fig. 3). Interestingly, this behavior appeared to be independent of the order in which the ranges were presented.

To quantify these trends, we determined how the target-response gain depended on target range by fitting Equation 6 through the data for each of the eight subjects, each of the three block sequences and for both dimensions (elevation vs azimuth). Three regression lines are highlighted, for subjects S6 (high-gain intercept, red), S1 (low-gain intercept, blue), and S5 (intermediate-gain intercept, yellow) for the elevation data. For the elevation response components, S6 had a gain intercept (β_0_ in Eq. 6; Fig. 4, filled circles on ordinate) of approximately β_0_ = 1.6, which decreased to a gain of *g_180_* = 1.1 for the large target range due to a negative gain slope (β_1_= –0.5; *g_180_* = β_0_–β_1_). In contrast, S1 had a low initial gain of only β_0_ = 0.5, which increased to *g_180_* = 0.8 (β_1_ = +0.3). Finally, subject S5 adjusted the response gain from β_0_ = 1.0 to g_180_ = 1.1 (i.e., β_1_ = +0.2). For the azimuth components, we highlighted three different subjects: S3 with a high-gain intercept, S7 with a low-gain intercept, and S8 with an intermediate response-gain intercept. The same trends in the gain changes toward the largest target range can be observed as for the elevation data: when the gain intercept was high, the gain tended to decrease across the larger target ranges; when the gain intercept was low, the gain increased as the target range expanded, whereas the response gain remained roughly constant for intermediate-gain intercepts near β_0_ = 1.0. On average, the narrow-range gain in azimuth is higher than 1, indicating a typical response overshoot.

Thus, there was a large intersubject variability in gains for the lowest target range. The intersubject variability decreased strongly for the largest target range, for which the gains attained values that were clustered near 1.0.

**Figure 5.**
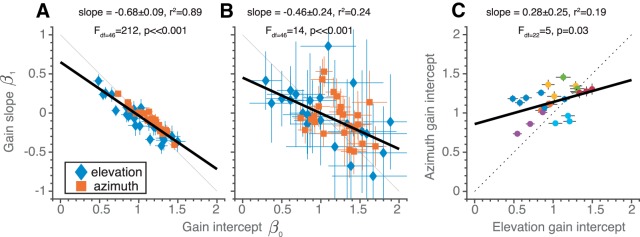
Gain change and narrow-range gain relationships in experiment 1. ***A***, Gain slope, β_1_, as a function of the gain intercept, β_0_ (Eq. 6), for both dimensions (azimuth and elevation, denoted in color), all three orderings (narrow-to-broad, broad-to-narrow, random), and all eight participants (*N* = 48). ***B***, Same analysis as in ***A***, performed for a selected target range of 30°, shared across all blocks ([–15, +15]° for azimuth and elevation). Results are qualitatively similar as in ***A***. ***C***, Gain intercepts for azimuth as a function of gain intercept for elevation. The various colors denote individual subjects. Colored symbols denote best-fit parameters, error bars indicate 95% confidence interval. Bold black lines denote the best fit simple linear regression line through the data. Dotted line in ***A*** and ***B*** indicates where data would lie if the broad-range gain equals 1. In ***C***, the dotted line indicates the x = y unity line.

Figure 5 quantifies this qualitative observation for all conditions, response components, and participants, by comparing the change in response gain over the 180° range (gain slope β_1_ in Eq. 6) with the gain intercept (β_0_ in Eq. 6). The very tight linear relationship, with *r^2^* = 0.89 (*p* ≪ 0.001), and a negative slope of –0.68, demonstrates that all subjects systematically adjusted their response gain, whenever they perceived a different target range. Importantly, the effect did not depend on the order in which the target ranges were presented. Instead, the gain adjustments depended on the idiosyncratic gain intercept, and was such that for the largest target range applied, the response gain approached a near-optimal value of *g* = 1.0. When the gain intercept was close to β_0_ = 1.0, the gain changed only little across the different target ranges (β_1_ ≈ 0). Although results are more variable, if we determine the gain slopes and intercepts for those locations which were presented in all blocks (i.e., for targets within the narrowest range), the same conclusions hold (Fig. 5*B*).

The gain intercepts for the azimuth and elevation response components were weakly correlated (*r^2^* = 0.19, *N* = 24, *p* = 0.03; Fig. 5*B*) and gain intercepts for the various block sequences (indicated by colors) tended to cluster. Thus, subjects with a high/low initial gain for one condition, also tended to have a high/low initial gain for other conditions.

### Sudden and steady adaptation

**Figure 6.**
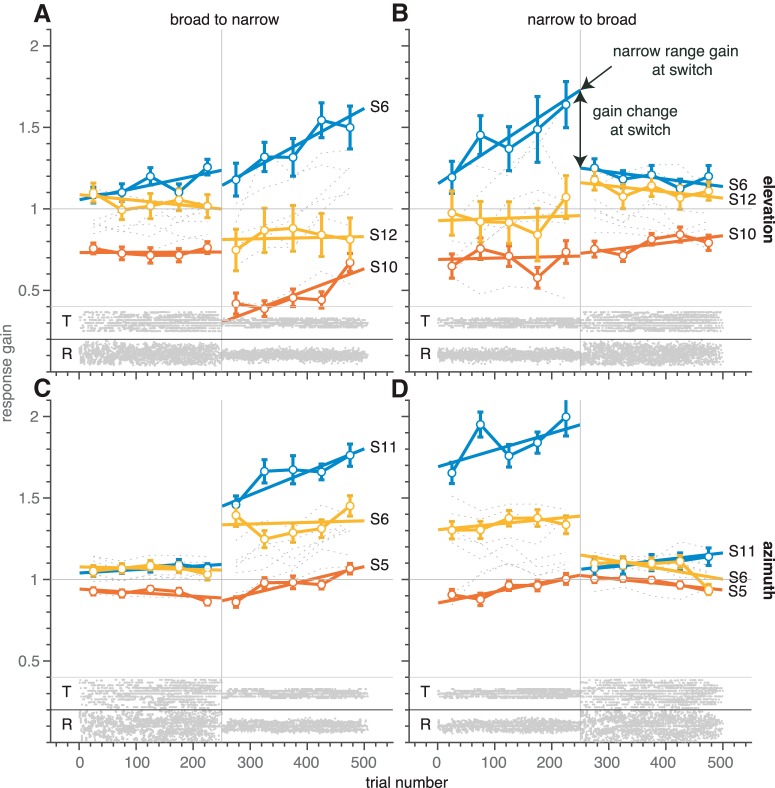
Gain dependence on target range and trial number in experiment 2. Ongoing response gains (top: elevation; bottom: azimuth) over the course of trials in experiment 2, in which the distribution switched from broad to narrow (***A***, ***C***), and from narrow to broad (***B***, ***D***) at trial 250 (vertical dashed lines and target-response distributions at the bottom). The horizontal dashed line indicates gain = 1. Note that the gains for the narrow target range are more variable across subjects than for the broad range. In addition, the variability in elevation gain for the broad range is slightly larger than for azimuth. Also, broad-range elevation gains are smaller than azimuth gains. Thin gray lines: windowed regression results (Materials and Methods). Connected colored open circles denote the localization gains for three representative subjects; error bars indicate the 95% confidence interval. Bold colored lines denote the best fit regression lines of Equation 8 through the data of these subjects.

We next tested whether the system would detect, and respond to, a sudden change in the target distribution, occurring within an experimental block of trials. In the second experiment, we therefore introduced an abrupt change from a narrow (50° range) to a broad (110° range) stimulus distribution, and vice versa, halfway the experimental run (after 250 trials). To follow the subjects’ response behaviors over time, we calculated the ongoing response gain in non-overlapping windows of 50 trials, throughout the experimental run of 500 trials (gray dotted lines for each of the 10 participants; Fig. 6; see Materials and Methods).

For both runs [broad-to-narrow (Fig. 6, left), and narrow-to-broad (Fig. 6, right)], the response gains across subjects had the smallest variability when subjects were confronted with the broad target range, whereas for the narrow target distribution the variability in response gains was much larger. This was true for both the elevation (Fig. 6, top) and azimuth (Fig. 6, bottom) components. Also, for the azimuth components alone, the narrow-range gains were often higher than 1 (Fig. 6*C*,*D*, narrow range), which is a clear violation of a strict interpretation of the error-minimization model described by Equation 2 (compare Fig. 1).

As in experiment 1, we show the highest-gain (blue), mid-gain (yellow), and lowest-gain (red) responder for the narrow target range to exemplify that this was predictive for the change in response gain after the switch in target range. The results suggest that when all gains were to be plotted from narrow to broad range (as in Fig. 4), by mirroring the data in the left-hand column with respect to trial 250, the curves would overlap to a large extent, except around the target-range switch, where the dynamics of the response changes become visible. The initial change in response gain to the switch was quite fast: within ∼50 trials subjects had adapted their gains to the new target range.

**Figure 7.**
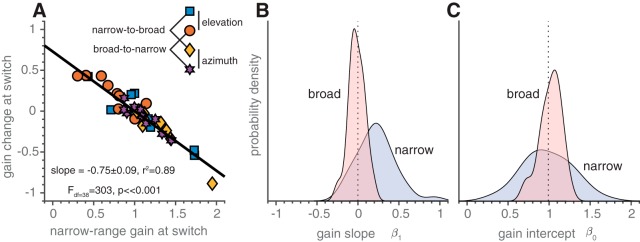
Gain change and narrow-range gain relationships in experiment 2. ***A***, Narrow-range gain as a function of the gain change at the switch (Fig. 6*B*). Note the high negative correlation between these quantities (compare Fig. 5*A*). Bold black line denotes the best fit linear relationship. Data are from ten participants, conditions, and response components (*N* = 40). Colors and symbols denote parameters from both narrow-to-broad and broad-to-narrow blocks and from both dimensions as indicated by the inset. Bold black line denotes the best fit simple linear regression line through the data. Dotted line indicates where data would lie if the broad-range gain equals 1. ***B***, Distributions of the gain slopes, β_1_, for the narrow and broad target ranges (Eq. 8). A slope around zero means that the gain did not change as a function of trial number. This was true, on average, for responses in the broad range. For the narrow range, however, the gains tended to increase. ***C***, Distributions of the gain intercepts, β_0_ (Eq. 8). Note the much wider distribution for the narrow target range.

Notably, the gain seemed to change slowly during the 250-trial epochs in which the target range was kept constant, especially during the narrow-range epoch. To quantify the fast and slow adaptive effects in this experiment, we estimated the initial gain at the first trial of a fixed target-range epoch (gain intercept, β_0_) and the change in gain during the epoch (gain slope, β_1_) through the regression analysis of Equation 8. This was applied separately to the two target-range epochs and both sequences (see bold colored lines for representative examples). From these parameters, we determined the narrow-range gain and the gain change at the switch (Fig. 6*B*, arrows). These show a high correlation (*r^2^* = 0.89, *N* = 40, *p* ≪ 0.001; Fig. 7*A*), indicating again (similar to the results in experiment 1; Fig. 5*A*) that the large variability in narrow-range gains is reduced in the broad-range epochs to an optimal value near 1. Also, if we repeat the analysis only for those locations presented in both blocks (i.e., for targets within the narrowest range), the same approximate results hold (slope = –0.38 ± 0.15, *r^2^* = 0.42, *F*_df=38_ = 27, *p* ≪ 0.001).

As noted above (and observed in Fig. 6), the change in gain during an epoch in which the target range was fixed varied between the narrow and the broad epochs (Fig. 7*B*). The gain slopes varied around 0 in the broad range (i.e., no overall gain change during this epoch; *t* test, *p* > 0.05; Fig. 7*B*, pink) while there was more variation in narrow-range gain slopes as indicated by a broader distribution, that also peaked at a value near 0.2 (*t* test, *p* < 0.001; Fig. 7*B*, purple). This indicates a steady increase in gain over trials for the narrow-range epoch. In line with this, the gain intercepts for the narrow target range are much more broadly distributed than the broad-range initial gains (Fig. 7*C*).

### Adaptation to dynamic changes

The results from the first two experiments demonstrate that listeners rapidly adjust their response gain to the perceived target range. In these experiments, the target ranges were kept fixed during a block of trials. We wondered whether these gain adjustments would also occur when the target range constantly changed, trial-by-trial. In the third experiment, stimulus locations were drawn from dynamically changing spatial distributions, in a harmonic way between a ΔT = 30° and ΔT = 120° range in azimuth and elevation, at one of four different repetition periods (*P* = 50, 100, 200, or 400 trials, respectively; see Materials and Methods; Eq. 3). The block started either with a broad (φ = 0), or with a narrow (φ = π) target distribution.

**Figure 8.**
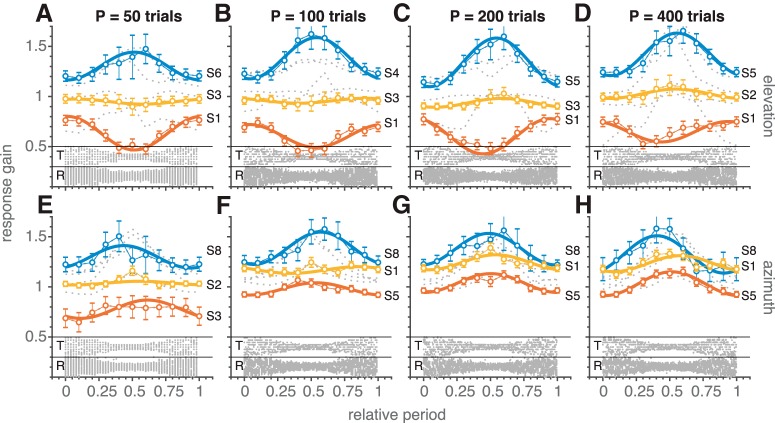
Gain dependence on modulating target range in experiment 3. Dynamic response-gain adjustments according to Equation 10 for all subjects (gray dotted lines: windowed analysis) and periods (*P* = 50, 100, 200, and 400 trials). Connected colored open circles denote the localization gains for three representative subjects; error bars indicate the 95% confidence interval. Bold colored lines denote the best fit regression lines of Equation 10 through the data of these subjects. The bottom of each panel shows target (T) and response (R) distributions (gray dots), pooled across subjects. Note opposite behavior of response gains for the low- vs. high-narrow gain responders in elevation (top row). The azimuth responses (bottom) are more similar across subjects, as the lowest narrow-range gains remained closer to one.

To analyze the data, we wrapped all responses onto a single full period of the trial distribution for φ = 0 (broad-narrow-broad) and phase-shifted the responses from the φ = π condition by –π radians. We then performed windowed analyses over 40-trial epochs, and the dynamic linear regression analysis of Equation 10 (see Materials and Methods). Figure 8 shows the results of these analyses for the dynamic response gains of this experiment during a full period. The target and response distributions are shown below each panel (same format as in Fig. 6). In each panel we highlighted three subjects, according to their narrow range gain (from Eq. 10, this amounts to β_0_ – β_1_): low, medium, and high narrow range gain. In line with the previous two experiments, the response gains across subjects varied much more for the narrow target range of 30°, when compared to the broad range of 120°. For the latter range, the gains scattered around the value of 1.0, both for the elevation components (top row), and for the azimuth components (bottom row). For the narrow range, azimuth and elevation gains (Fig. 8*E–H*) were often higher than 1. During the dynamic change toward the narrow target range (around the center of each panel) the elevation gains systematically increased (upper black lines), stayed approximately constant (middle black lines), or decreased (lower black lines), to return to their initial broad-range values at the end of the period. These patterns remained quite similar for the four different periods (50, 100, 200, and 400 trials, respectively), and across subjects. For the azimuth response components, we obtained a similar behavior, albeit that the variation in gain for the narrow range was smaller than for elevation, and that the absolute gains attained higher values. As a result, the azimuth gains always decreased from the narrow range to the broad range.

**Figure 9.**
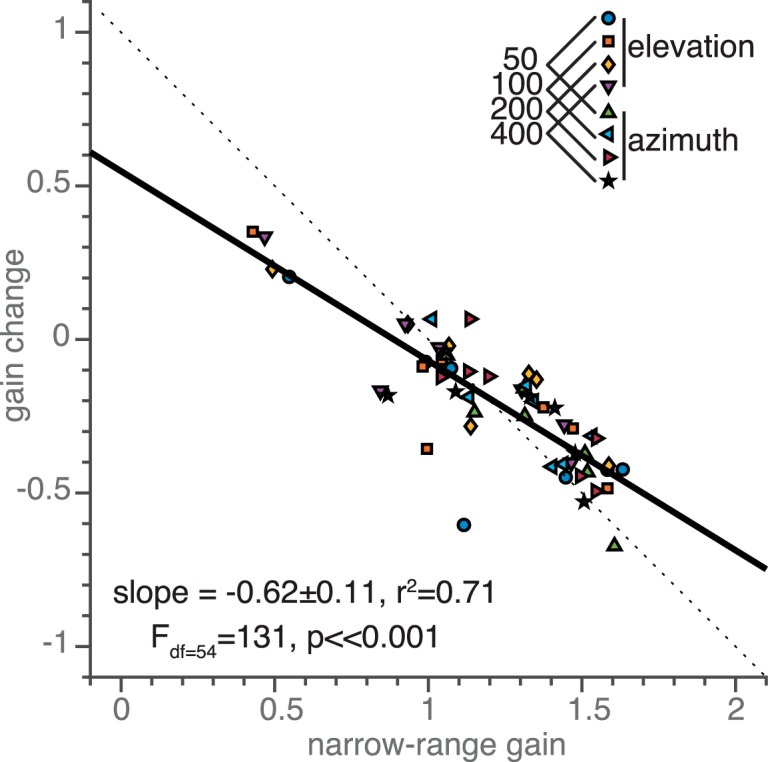
Gain change and narrow-range gain relationships in experiment 3. Gain change (2β_1_) as a function of the narrow-range gain (β_0_–β_1_) for the results of experiment 3 (Eq. 10). Colored symbols denote data from seven participants, four periods, and two response components (*N* = 56) as indicated by the inset. Bold black line denote the best fit simple linear regression line through the data. Dotted line indicates where data would lie if the broad-range gain equals 1.

Figure 9 quantifies the relationship between the narrow-range gain and the change in gain across the target-range period (given by Δg = 2β_1_; see Materials and Methods). In line with the observations in Figure 8, when the narrow-range gain was high (>1), the response gain decreased (Δg < 0), and when it was low (<1) it tended to increase (Δg > 0) with a high correlation (*r^2^* = 0.71 and *p* ≪ 0.001). In addition, the slope of this relationship (slope = –0.62) is of similar magnitude for experiments 1 (slope = –0.68; Fig. 5*A*) and 2 (slope = –0.73; Fig. 7*A*), also if targets are selected within the narrowest range only (slope = –0.53 ± 0.19, *r^2^* = 0.35, *F*_df=54_ = 30, *p* ≪ 0.001).

## Discussion

### Summary

We studied human sound-localization to targets drawn from different spatial distributions. Head-orienting responses were made under open-loop conditions, as subjects never received feedback about their performance. We reasoned that if subjects rely only on immediate acoustic cues, the response gain should be independent of trial history and spatial target distribution. In contrast, if the system collects non-acoustic evidence from previous trials to optimize its response strategy, the spatial target distribution could potentially influence response behavior.

Subjects were indeed sensitive to the spatial range of sounds. We found highly idiosyncratic stimulus-response gains for narrow spatial distributions which deviated from a strict error-minimization model (as described in Eq. 2 and Fig. 1). However, when stimuli were drawn from a broad spatial range, intersubject variability decreased substantially, and response gains clustered around an optimal gain of one (Figs. 4, 6, and 8).

### Idiosyncratic behavior

**Figure 10.**
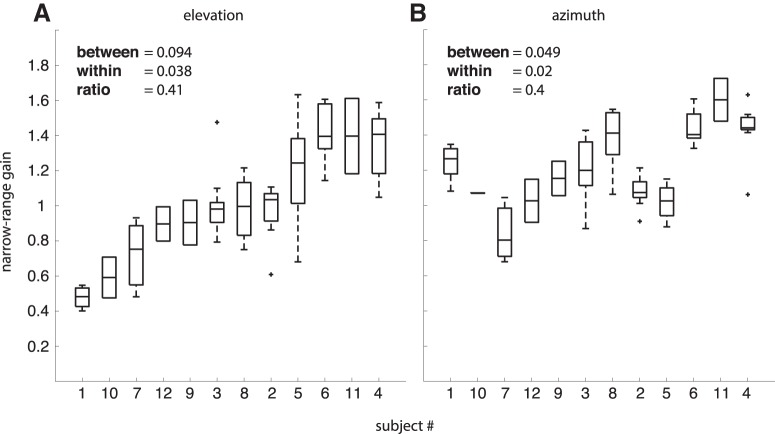
Variability in narrow-range gains. ***A***, Subjects ordered according to the median of their elevation gains. The ratio between intrasubject and intersubject variability is 0.41. ***B***, Same subject ordering as in ***A*** for azimuth. The ratio is 0.40. Note that elevation and azimuth results are positively correlated (*r* = 0.48, *N* = 72, *p* < 10^−5^; compare Fig. 5*C*). The median, the 25th and 75th percentiles, the most extreme datapoints not considered to be outliers, and the outliers are indicated by the central mark, the edges of the box, whiskers and plus-symbols, respectively.

Although response gains for blocks with narrow target ranges were idiosyncratic, they were quite consistent within subjects. Note that data within an experiment were collected on different days, whereas experiments 1–3 were conducted over a period spanning four months. However, subjects responding with low/high gain for the narrow target range in experiment 1, also tended to do so in experiments 2 and 3. Figure 10 summarizes the subject-specific narrow-range gains for elevation (Fig. 10*A*) and azimuth (Fig. 10*B*), ranking subjects according to the median of their elevation gains. Clearly, within-subject variability is much smaller than between subject variability: the ratio within/between was ∼0.4 for both coordinates.

### The sound-localization problem

Sound localization results from a neuro-computational process, which compares binaural inputs (ITDs and ILDs) to determine azimuth and extracts monaural spectral pinna cues (HRTFs) to estimate elevation. Still, even a single broadband sound cannot provide unique spatial information, as the elevation-dependent spectrum at the eardrum, *S(f; ε),* results from multiplying source spectrum, *X(f)*, with the elevation-dependent pinna filter: *S(f; ε) = HRTF(f; ε)⋅X(f)*. Since both are a priori unknown to the auditory system, sound localization is mathematically “ill-posed” (Middlebrooks and Green, 1991; Hofman and Van Opstal, 1998): infinitely many combinations of source spectra and HRTFs could generate the same sensory spectrum.

Thus, the auditory system needs additional information to infer the most likely source elevation. We showed previously that if the system assumes that (1) HRTFs are unique for each elevation, and (2) source spectra do not resemble any HRTF, spectral cross-correlation of the sensory spectrum with all stored HRTFs can identify the veridical source elevation by maximum likelihood estimation (Hofman and Van Opstal, 1998). In this way, sound localization can be accurate, and relatively robust to the sound’s spectral shape (Kulkarni and Colburn, 1998).

The HRTFs may be learned through exposure to different acoustic environments, combined with sensorimotor feedback (Goossens and Van Opstal, 1997). For example, the auditory system adapts to acute HRTF changes (Hofman et al., 1998; Van Wanrooij and Van Opstal, 2005), and to slow changes due to age-related pinna growth (Otte et al., 2013). Presumably, the system acquires spatial information by interacting with sounds in daily life, using visuomotor and sensorimotor error feedback (Shinn-Cunningham et al., 1998; Zwiers et al., 2003; Carlile et al., 2014). However, because of the inherently ill-posed nature of the problem, the system can never be sure about the true sound direction. It may hence rely on statistical inference to estimate the most likely target location at the lowest cost. The underlying neural mechanisms, however, have so far not been identified.

### Ecological range

In the natural environment, sounds could originate from all around. As such, laboratory stimuli with a limited spatial range might appear non-ecological. However, it should be noted that the major sound-localization cues (the ITDs and ILDs) in natural recordings scatter around 0 because people tend to face the person they communicate with. Moreover, recordings also show that the majority of natural sounds originate from a limited range in elevation, and that the human sound-localization system may have adapted to these features (Parise et al., 2014). Moreover, under natural conditions, subjects will typically use multiple sensory signals (visual, auditory, vestibular, motor), which all need to be centrally integrated to form coherent spatial-temporal percepts of objects in the environment (Stein and Stanford, 2008; Van Wanrooij et al., 2010; Van Grootel et al., 2011; Van Barneveld and Van Wanrooij, 2013). For adequate audiovisual integration it should be noted that the visual range is limited to only a narrow frontal domain, which again suggests that many natural sound-localization behaviors will be performed within this range too.

In our experiments, all sounds had broad-band flat spectra, and as such were well-localizable, although they were presented under fully open-loop conditions in total darkness, without any exogenous feedback. This is further evidenced by the very high correlation coefficients and consistent response behaviors within subjects, and across tasks, listening conditions, and stimulus ranges. Because sounds were broadband, they never induced localization ambiguities, such as front-back confusions (which would show up as bimodal response distributions). It is therefore hard to imagine that these highly consistent, stimulus-related results could reflect a non-relevant response behavior, elicited by non-ecological stimuli.

### Related work

Many studies have demonstrated response adaptation to changes in the environment. Most studies used explicit (visual) feedback to influence response behavior. For example, manipulation of the perceived errors of eye-hand control through noisy visual feedback showed that the brain derives the underlying error distribution across trials through Bayesian inference (Körding and Wolpert, 2004). The Bayesian formalism also extends to audiovisual integration (Körding et al., 2007), movement planning (Hudson et al., 2007), ventriloquism (Alais and Burr, 2004), visual speed perception (Stocker and Simoncelli, 2006), and auditory spatial learning (Carlile et al., 2014). Furthermore, it may explain learning of the underlying distribution of target locations in a visual estimation task (Berniker et al., 2010). Also, sound-localization behavior adapts to chronic and acute changes in the acoustics-to-spatial mapping (Hofman et al., 1998; Shinn-Cunningham et al., 1998; Zwiers et al., 2003; King et al., 2011; Otte et al., 2013; Carlile et al., 2014).

Minimizing the MAE, as described in the Introduction (Eq. 2), is mathematically equivalent to the optimal Bayesian decision rule on Gaussian distributions that selects the maximum of the posterior distribution (the maximum-a-posteriori, or MAP strategy (Körding and Wolpert, 2004; Ege et al., 2018):

(11) POST(ε*|ε)∝L(ε|ε*)·P(ε*) and R=maxarg[POST(ε*|ε)]

with L(ε|ε*) the likelihood function of the noisy sensory input for a target presented at ε*, with uncertainty, σ*_T_*; P(ε*) is the prior distribution, or expectation, of potential target locations, and *R* is the selected MAP response. For a fixed prior, the MAP strategy provides an optimal trade-off between mean absolute localization error (accuracy) and response variability (precision). For Gaussian distributions, the MAP rule predicts that the stimulus-response gain depends on the sensory noise, σ*_T_*, and the prior width, σ*_P_,* by:

(12) $$g\equiv \frac{R}{\varepsilon *}=\frac{\sigma_{P}^{2}}{\sigma_{P}^{2}+\sigma_{T}^{2}}$$

Recently, we (Ege et al., 2018) found that for a fixed target range, the human sound localization system might indeed rely on such a Bayesian decision rule, as the results indicated that the localization gain *g* depended on the sensory noise, σ*_T_* in a systematic fashion.

In our current experiments, the prior width may have varied with the expected target range: σ*_P_ =* σ*_P_(ΔT)*. The idiosyncratic differences in initial gains, observed in this study, could thus be partially due to idiosyncratic differences in initial priors. The present study challenged the auditory system to update its prior only on the basis of endogenous signals.

Several studies have shown that the auditory system rapidly adapts to the statistics of environmental acoustics, without overt exogenous feedback. For example, neurons in inferior colliculus (IC) of anesthetized guinea pigs shift their sound-level tuning curves according to the mean and variance of sound levels (Dean et al., 2005). Interestingly, these rapid adjustments already manifest at the auditory nerve (Wen et al., 2009). Likewise, ILD tuning of IC neurons in anesthetized ferrets adjusts to the ILD statistics of dichotic sounds, while these same stimuli induce perceptual shifts to ILD sensitivity in humans (Dahmen et al., 2010). Finally, it has been shown that head-orienting reaction times to audiovisual stimuli depend systematically on trial history, and on the probability of perceived audiovisual spatial alignment, without providing exogenous feedback (Van Wanrooij et al., 2010).

### Potential neural mechanisms

The present study demonstrates that the auditory system continuously evaluates its localization performance on the basis of present and (recent) past trial information, and of its own responses, even without any exogenous feedback. We hypothesize that the system may have used two sources of endogenous information: (1) if kept in memory, the perceived acoustic cues implicitly inform the system about the current probability distribution of estimated source locations, and (2) efference copies, together with proprioceptive information from neck muscles and vestibular responses, yield behavioral information about its goal-directed head-orienting responses, and hence about the system’s own localization estimates and errors. Earlier studies have revealed that the auditory system indeed incorporates static and dynamic eye and head orientations to estimate sound locations (Goossens and Van Opstal, 1999; Vliegen et al., 2004).

We conjecture that by combining these information sources, the brain could estimate the expected mean localization error (Eq. 2) as its performance cost. To minimize this cost, the response gain should depend systematically on the perceived target range, which is qualitatively supported by our data. Quantitatively, however, the data seem to differ from the predictions. First, although Equation 2 predicts gains <1.0 (Fig. 1*A*), we obtained slightly higher response gains for the largest target ranges. Second, the large idiosyncratic variability of narrow-range response gains (see above in the Results section, e.g. Fig. 5, 7, and 9) seem not in line with minimizing a cost function.

However, both model deviations might actually be expected for several reasons. First, it should be noted that it is impossible to assess the actual internal estimates of the different components underlying the cost of Equation 2. (1) The actual perceived target range depends on internal mappings of weighted ITD, ILD and spectral cues onto source locations. (2) The head-motor response involves a sensorimotor transformation from cue-derived sensory percept to motor output with inherent uncertainty. (3) Internal noise sources of the sensorimotor transformations are not directly accessible. These different components are not independent and combine in a nonlinear way to the cost. As a result, measured gains of stimulus-response relations may not exactly correspond to internal estimates of the system’s own optimal gains, described by Equation 2.

Further, the actual strategy of the auditory system might be to keep the cost within certain bounds around the minimum, as the target range itself is at best an internal estimate, endowed with uncertainty of its own. The simulations show that for a small (perceived) target range, the tolerance could be substantial, as the effect of gain changes on the mean absolute error is quite modest. For example, Figure 1*A* shows that when the gain would vary between 0.1 and 0.8, the mean error would change by merely 1.5°, which remains within the spatial resolution of the human auditory system. Similarly, for gains higher than 1 the mean error would also increase only slightly for the narrow target range. In lieu of that, the observation that the gains for the azimuth components are typically higher (not lower) than 1 is interesting. This might suggest that overshooting the targets is a better strategy than undershooting, although both strategies would yield the same sub-optimal error. In natural environments, this would make sense, as an overshooting strategy would allow for exploratory behavior even when sensory evidence would be poor.

In contrast to the effects for the narrow target range, if the same gain change occurs for the largest target range, the mean error would vary by >15°. This strong range-dependent effect on the cost could explain the observed idiosyncratic variability at the small target ranges (Figs. 4, 6, 8, 10), the slow gain changes seen during prolonged exposure to narrow target ranges (Fig. 6, 7*B*), as well as the inverse relationships that pull response gains toward near-optimal values around 1.0, with limited idiosyncratic variability, for the wider target ranges (Figs. 5, 7, 9).

## Synthesis

Reviewing Editor: Darcy Kelley, Columbia University Decisions are customarily a result of the Reviewing Editor and the peer reviewers coming together and discussing their recommendations until a consensus is reached. When revisions are invited, a fact-based synthesis statement explaining their decision and outlining what is needed to prepare a revision will be listed below. The following reviewer(s) agreed to reveal their identity: Andrew Dykstra, Shuang Li.

The authors measured sound localization in human listeners via head turning in response to free-field stimulation in a dark, anechoic room and investigated how listeners adjust the range of their motor output with respect to the range of target inputs in the absence of explicit feedback. They found that listeners indeed adjust their target-response gain for differing target ranges, such that they have a near-ideal (i.e. unity) gain when the range of target stimuli is spatially extended but idiosyncratic (i.e. listener-specific but consistent with a listener) behavior for smaller target ranges.

In general, this is an interesting study that could contribute to our understanding of both sound localization and particularly sensorimotor integration. However, the statistical analysis of the data, the clarity of its presentation and issues of interpretation requires substantial revisions before re-consideration for publication as enumerated below:

Statistical analyses:

1. What is the confidence interval of the estimation of g? Confidence interval of gain estimation should be taken into consideration when interpreting the experimental results.

2. Quantitative criteria for significance of the linear regression (p value) should be provided. A high r^2 does not guarantee the existence of linear relationship.

3. The mean of gain across subjects did not change with different target range, it was the variability of the gain that changed. Thus, the conclusion in line 316 - 317: “As a result, subjects systematically decreased their response gain with increasing target range...” is not correct.

4. For Figure 4, it is not clear what criteria allowed replicable (i.e. non-arbitrary) separation of groups into high, low, and intermediate gain intercepts. As the data in Figure 4 are heteroscedastic, possible confounding variables should be taken into consideration.

Data presentation:

1. More use of color in some of the figures, particularly Figures 4, 6, 7, and 8, would aid in data presentation of the data. In Figures 4, 6, and 8, color would make it easier to identify individual subjects (i.e. specific gray lines) and their associated modeled gain adjustments (i.e. associated black lines). In Figure 7, color would make it easier to identify the different conditions (particularly in panel A).

Interpretation:

1. There seems to be a discrepancy between the data and discussion of the results, on one hand, and the conclusions the authors draw in the abstract and significance statement, on the other. In the abstract and significance statement ate that results are explained by a model in which overall absolute error is minimized. The introduction includes a model that suggests a mapping from response gain to mean absolute error for different target ranges, with ‘optimal’ response gain decreasing for increasingly smaller target ranges. However, this is not what the data show. Instead, as is pointed out, listener behavior at these smaller (non-ecological, see point below) target ranges was idiosyncratic, with some listeners undershooting the real target range and others overshooting it. A better supported conclusion is that that listeners do indeed adjust the gain of their target-response mapping for different target ranges and that this behavior gets closer to optimal the larger the range is.

2. Especially in audition, sounds with such a limited spatial range appear non-ecological. Some discussion of how this factor might have impacted results is warranted.

3. The relative consistency (across subjects) of azimuth gain adjustments in the sinusoidal paradigm of experiment 3 (fig. 8) was striking. In particular, response gains tended to be higher for smaller target ranges (and even mostly > 1), with the exception of S1 (who, as the authors note, was the only subject who was not naive to the purpose of the study) and perhaps S3 for elevation. This results appears opposite to the direction of the optimal gain adjustment expected from the model (Fig. 1). Might these data be more consistent with a simpler model in which listeners maximized the differentiability of responses? For example, for smaller target ranges, sujects might feel free to use a larger response range to explicitly differentiate between more probable finer-grained differences in the input. This possible interpretation might be examined (at least for azimuth) by computing gain fits in the context of large target ranges but using only the data from the smaller range trials (e.g. the 120- or 180-degree blocks of experiment 1 and the large-range sections of experiments 2 and 3. Listeners might have a compressive non-linearity for their target-response functions such that they consistently overshoot slightly eccentric azimuthal targets but are more accurate for more eccentric targets. This might not be true for elevation, where the narrow-range gains are much more variable, but might be true for azimuth, where most subjects showed gains larger than 1 for narrow ranges.

In general, unnecessary claims of novelty (e.g. ‘for the first time’) should be avoided. More conceptual terms and citations of previous work instead of equations should be presented in the Introduction.

Minor concerns:

1. Line: 32, justify why using the mean absolute localization error instead of other error format

2. Line 55 - 58: citation for definitions in equation (1) and (2). Why i the relationship in (1) linear? What is the physiological basis of the linear relationship?

3. Line 76: provide details of the model simulation.

4. Line 168: For each experiment, how many repeated measure were performed per human subject? If there were repeated measure, how did were data analyzed and gain terms obtained?

5. Line 184: the large overlapping between ΔT = 120 deg and 160 deg would not seems to t justify the pooling of the data.

6. Line 191: define ‘sessions’.

7. Figure 2: Color results from elevation and azimuth

8. Figure 5: Formats for figure legends and text should be consistent; a careful grammar review is required.
